# A Necroptosis-Related lncRNA-Based Signature to Predict Prognosis and Probe Molecular Characteristics of Stomach Adenocarcinoma

**DOI:** 10.3389/fgene.2022.833928

**Published:** 2022-03-07

**Authors:** Lianghua Luo, Leyan Li, Li Liu, Zongfeng Feng, Qingwen Zeng, Xufeng Shu, Yi Cao, Zhengrong Li

**Affiliations:** ^1^ Department of Gastrointestinal Surgery, The First Affiliated Hospital, Nanchang University, Nanchang, China; ^2^ Queen Mary School, Medical Department of Nanchang University, Nanchang, China

**Keywords:** necroptosis, lncRNA, signature, prognosis, molecular characteristics, stomach adenocarcinoma

## Abstract

**Background:** As a caspase-independent type of cell death, necroptosis plays a significant role in the initiation, and progression of gastric cancer (GC). Numerous studies have confirmed that long non-coding RNAs (lncRNAs) are closely related to the prognosis of patients with GC. However, the relationship between necroptosis and lncRNAs in GC remains unclear.

**Methods:** The molecular profiling data (RNA-sequencing and somatic mutation data) and clinical information of patients with stomach adenocarcinoma (STAD) were retrieved from The Cancer Genome Atlas (TCGA) database. Pearson correlation analysis was conducted to identify the necroptosis-related lncRNAs (NRLs). Subsequently, univariate Cox regression and LASSO-Cox regression were conducted to establish a 12-NRLs signature in the training set and validate it in the testing set. Finally, the prognostic power of the 12-NRLs signature was appraised via survival analysis, nomogram, Cox regression, clinicopathological characteristics correlation analysis, and the receiver operating characteristic (ROC) curve. Furthermore, correlations between the signature risk score (RS) and immune cell infiltration, immune checkpoint molecules, somatic gene mutations, and anticancer drug sensitivity were analyzed.

**Results:** In the present study, a 12-NRLs signature comprising REPIN1-AS1, UBL7-AS1, LINC00460, LINC02773, CHROMR, LINC01094, FLNB-AS1, ITFG1-AS1, LASTR, PINK1-AS, LINC01638, and PVT1 was developed to improve the prognosis prediction of STAD patients. Unsupervised methods, including principal component analysis and t-distributed stochastic neighbor embedding, confirmed the capability of the present signature to separate samples with RS. Kaplan-Meier and ROC curves revealed that the signature had an acceptable predictive potency in the TCGA training and testing sets. Cox regression and stratified survival analysis indicated that the 12-NRLs signature were risk factors independent of various clinical parameters. Additionally, immune cell infiltration, immune checkpoint molecules, somatic gene mutations, and half-inhibitory concentration differed significantly among different risk subtypes, which implied that the signature could assess the clinical efficacy of chemotherapy and immunotherapy.

**Conclusion:** This 12-NRLs risk signature may help assess the prognosis and molecular features of patients with STAD and improve treatment modalities, thus can be further applied clinically.

## Introduction

Gastric cancer (GC) is a highly aggressive and extremely heterogeneous malignancy that causes high morbidity and mortality globally. According to *Global Cancer Report 2020*, GC accounts for over 1 million new cases and nearly 760,000 deaths worldwide each year (Sung et al., 2021). The most general histological subtype of GC is stomach adenocarcinoma (STAD), which is highly fatal. Despite substantial advances in surgical procedures, adjuvant treatments, and examination techniques over the past decades, the prognosis of patients with STAD remains poor, especially for those in the advanced stage with a 5-years survival rate below 20% (Ferlay et al., 2019; Tan, 2019). Due to differences in genetic characteristics, STAD patients with similar tumor histology, and the same pathological stage may have completely distinct oncologic outcomes. Hence, exploring novel efficient biomarkers and therapeutic approaches is crucial to improving the prognosis and treatment of STAD patients.

Necroptosis is a new type of cell death mediated by Receptor Interacting Protein Kinase1/3 (RIPK1/RIPK3) and executed by Mixed Lineage Kinase Domain Like Pseudokinase (MLKL) (Declercq et al., 2009; Marshall and Baines, 2014). The exact effect of necroptosis in tumors is complex and controversial. On the one hand, necroptosis suppresses tumors during cancer initiation and metastasis by inhibiting osteosarcoma, colorectal cancer, acute myeloid leukemia (AML), and breast cancer (Fu et al., 2013; Nugues et al., 2014; Feng et al., 2015; Koo et al., 2015). On the other hand, necroptosis has been demonstrated to promote tumor cell growth and tumor metastasis. *In vivo*, different RIPK1 inhibitors can prevent tumor growth and limit metastasis (Strilic et al., 2016; Li et al., 2018). In a mouse breast cancer model, the absence and depletion of MLKL blocked metastasis of breast cancer cells to the lung (Jiao et al., 2018).

Long non-coding RNA (lncRNA) is a class of non-protein-coding RNAs (ncRNAs) with a length of over 200 nucleotides but several special functions, such as mRNA splicing, transcription regulation, and mRNA post-transcriptional regulation (Guttman and Rinn, 2012; Zuo et al., 2021). LncRNAs have been proved closely related to tumor development, metastasis, and cancer immunity, and their good molecular stability could make them new potential prognostic biomarkers in cancer patients (Huarte, 2015; Bhan et al., 2017; Denaro et al., 2019; Statello et al., 2021). Therefore, further elucidating the relationship between necroptosis-related lncRNAs (NRLs) and STAD is essential for exploring novel therapeutic targets for STAD and improving patient prognosis.

In this study, NRLs were mined from TCGA-STAD transcript data via Pearson correlation analysis. Then, a 12-NRLs signature for predicting the survival of STAD patients was proposed through univariate analysis and LASSO regression analysis. Moreover, molecular characteristics exploration based on the 12-NRLs signature was crucial to supplying a robust theoretical basis for applying immunotherapy and chemotherapy to STAD patients.

## Materials and Methods

### Data Collection

The clinical information and RNA-sequencing (RNA-seq) profile of STAD patients were retrieved from the TCGA platform (https://portal.gdc.cancer.gov/repository , up to 13 September 2021). The RNA-seq data from the TCGA database had been normalized to Fragments Per Kilobase Million (FPKM) format for subsequent data analysis. To improve research accuracy, only samples with integral clinicopathological information were included for analysis after pre-processing based on the Perl programming language (version Strawberry-Perl-5.30.0.1; https://www.perl.org). Subsequently, all patient information was randomly allocated into the training and the testing sets via the “caret” package in R (version 4.1.0, https://www.r-project.org/). A total of 39 necroptosis-related genes was acquired by searching published studies (Hanson, 2016; Chen et al., 2019; Molnár et al., 2019; Zhu et al., 2019; Liu et al., 2021). In addition, the mutation data of STAD samples were downloaded in MAF format from the TCGA database.

### Identification of NRLs in STAD

Using the GTF annotation files of human lncRNAs retrieved from the GENCODE (https://www.gencodegenes.org/ , up to 13 September 2021) website, 4,497 lncRNAs were identified from the TCGA-STAD RNA-seq data. Subsequently, the co-expression relationships between necroptosis-related genes and all lncRNAs in STAD samples were examined by Pearson correlation analysis, and those lncRNAs were considered significantly associated with necroptotic mRNAs. |Coefficient| > 0.3 and *p*-value < 0.01 were considered as the cutoff.

### Establishment of the Necroptosis-Related lncRNA-mRNA Co-expression and Protein-Protein Interaction Network

To demonstrate the mutually regulated connection between NLRs and corresponding target mRNAs, we visualized the lncRNA-mRNA network using the “igraph” package in R. Additionally, the necroptosis-related genes regulated by candidate lncRNAs were uploaded to the STRING (version 11.5, https://www.string-db.org/) website to build the protein-protein interaction (PPI) network.

### Construction and Verification of the NRLs Signature of STAD

Univariate Cox proportional regression analysis was applied to determine the prognosis-related lncRNAs (*p* < 0.05) in TCGA-STAD. Then, the LASSO Cox regression algorithm was adopted to ascertain the optimal panel of prognostic lncRNAs and establish an optimal signature. Next, each STAD patient’s survival risk score (RS) was calculated based on the standardized expression levels of NRLs and the corresponding regression coefficients derived from the LASSO regression analysis. The calculation is as follows: RS = $\sum_{i=1}^{n}Coef\left(i\right)\times x\left(i\right)$ , where *Coef (i)* and *x(i)* represent the coefficient and the standardized expression levels of each NRLs, respectively. The median RS of the training set was utilized as the demarcation point to categorize all included STAD samples into the low-risk or high-risk subsets. Kaplan-Meier (K-M) curves were adopted to contrast the overall survival (OS) of the high-risk and low-risk subsets in the training and testing sets using the “survival” package. The time-dependent ROC curves were used to evaluate survival prediction, and areas under the ROC curve (AUC) were calculated to assess the predictive accuracy and specificity of the NRLs signature.

### Nomogram Establishment and the Correlation Between the Prognostic Signature and Clinicopathological Characteristics

In order to verify the independence of the NRLs signature, univariate analysis and multivariate Cox regression analysis were conducted to evaluate the relationship between clinical features or the NRLs signature with OS, especially to study whether the NRLs signature could be considered a hazard element independent of other clinicopathological factors, such as gender, age, grade, stage, and TNM stage for STAD patients. A stratified survival analysis based on different clinicopathological features was conducted to investigate the applicability of the NRLs-based signature. A nomogram considering independent prognostic factors was developed to predict the 1-, 3-, and 5-years OS rates of STAD patients via the “rms” package. In addition, the calibration curves were drawn to compare the consistency between the predicted 1-, 3-, and 5-years survival probability based on the nomogram and the actual observations. Finally, the decision curve analysis (DCA) curve was plotted to appraise the clinical effect of the nomogram by calculating the net benefits of a series of risk threshold probabilities using the “rmda” package.

### Immune Cell Infiltration Analysis

The components of the immune and stromal cells in the tumor microenvironment (TME) of each STAD sample were computed to verify the differences of microenvironment features between unequal risk groups using the ESTIMATE algorithm. The “CIBERSORT” package was adopted to extract the relative proportions of 22 types of human infiltrated immune cells, which could reveal the correlation between the risk signature and immune-cell characteristics. Additionally, the enrichment levels of 29 immune-related functions between the two sets were evaluated by Single sample Gene Set Enrichment Analysis (ssGSEA) scores.

### Pathway Enrichment Analysis

In order to clarify the differences of enriched pathways between the low-risk and high-risk subsets, the Gene Set Enrichment Analysis (GSEA) software (version 4.10) was utilized to carry out the Kyoto Encyclopedia of Genes and Genomes (KEGG) pathway enrichment analysis.

### Significance of the NRLs-Based Signature in Chemotherapy and Immunotherapy

In order to predict the response of STAD patients in the two different risk subsets to chemotherapy drugs, the “pRRophetic” package was used to assess the half-maximal inhibitory concentration (IC50) of 20 ordinary chemotherapy drugs highly recommended by the 8th American Joint Committee on Cancer (AJCC) guidelines, such as Paclitaxel, Cisplatin, and Imatinib. The differences in IC50 values between the high-risk and low-risk subsets were analyzed by Wilcoxon signed-rank test. The discrepancies of 5 immune checkpoint blockade-related molecules between low-risk and high-risk subsets were examined to appraise the predictive effect of the signature for STAD immunotherapy. The file of Tumor Immune Dysfunction and Exclusion (TIDE) score, T cell dysfunction score, and T cell exclusion score was retrieved from the TIDE website (http://tide.dfci.harvard.edu). To estimate the clinical response to immune checkpoint inhibitor (ICI) therapy for STAD patients, TIDE analysis was conducted to predict the potential effect of immunotherapy in different risk subsets.

## Results

### Identification of NRLs in STAD

**FIGURE 1:**
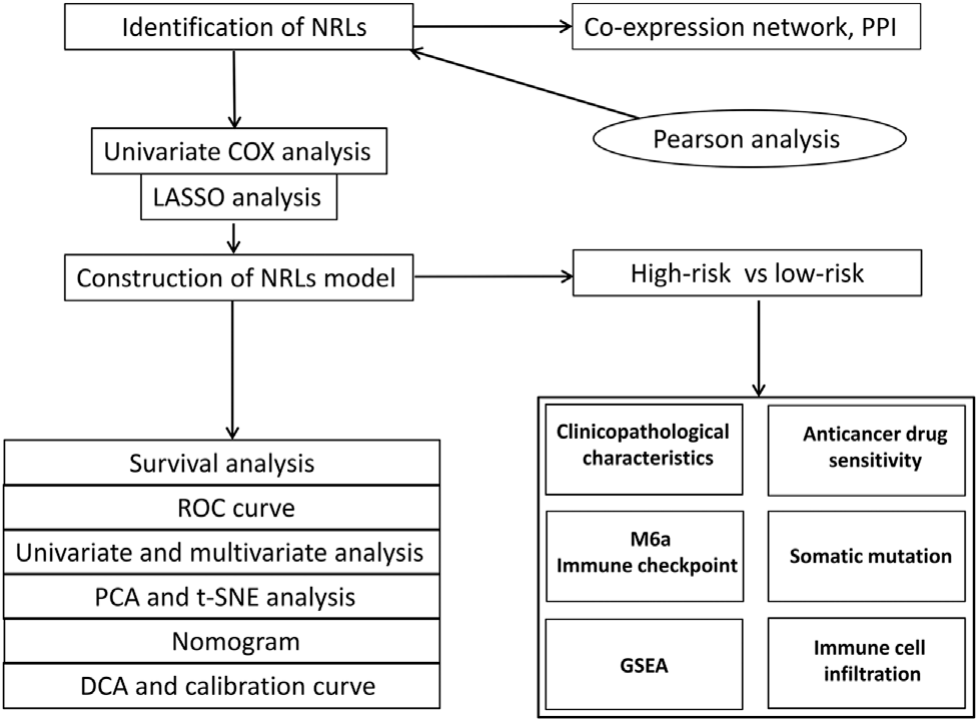
Research flow chart.

**FIGURE 2:**
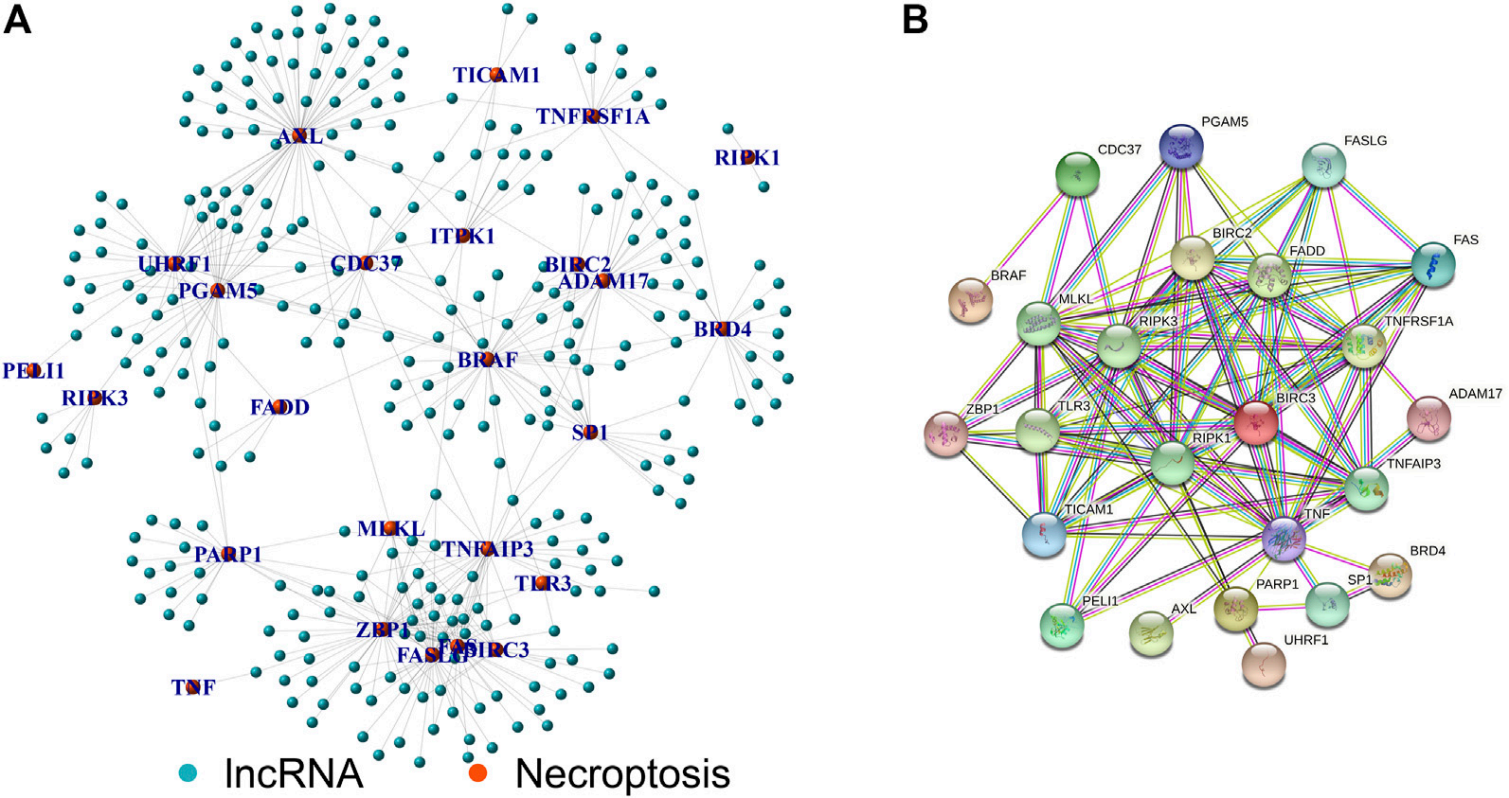
Establishment of lncRNA-mRNA co-expression and PPI network. **(A)** Necroptosis-related lncRNA-mRNA co-expression network diagram. **(B)** PPI network of necroptosis-related genes.

Figure 1 shows the flow chart of the research scheme. The transcriptome RNA-seq and clinical data of 407 STAD patients were collected from the TCGA database, which contained 375 samples of GC tissues and 32 samples of normal gastric tissues. Moreover, 368 samples with complete clinical information were picked out for the following analysis. According to the GTF annotation file of human lncRNAs, 4,497 lncRNAs were identified in TCGA-STAD gene expression files. A total of 39 necroptosis-related genes are exhibited in Supplementary Table S1, and 305 lncRNAs were ultimately selected as necroptosis-related lncRNAs. An mRNA-lncRNA co-expression network based on the Pearson correlation analytical results was established to determine the potential effects of NRLs (Figure 2A). A PPI network was also established to investigate the connection of these necroptosis-related genes using the STRING database (Figure 2B).

### Construction of the NRLs Prognostic Signature

**FIGURE 3:**
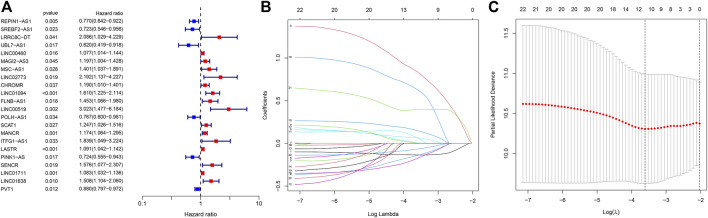
Univariate regression analysis and LASSO regression analysis. **(A)** The forest plot of prognostic-related lncRNAs. **(B)** LASSO coefficient profiles of necroptosis-associated lncRNAs. **(C)** The partial likelihood deviance with changing of log(λ). *:*p* < 0.05 **:*p* < 0.01 ***:*p* < 0.001.

The 368 STAD samples with lncRNAs expression data and integral survival information from the TCGA database were applied to determine the relationship between NRLs and prognosis. An RS-based prognostic signature was built for the final prognostic prediction. Firstly, univariate Cox proportional hazard regression analysis was employed to initially screen 22 lncRNAs associated with the OS of STAD patients from the 305 NRLs (*p* < 0.05, Figure 3A; Supplementary Table S2). Then, LASSO Cox regression analysis was conducted using the training set to acquire the lncRNAs with the highest prognostic value via the “glmnet” package (Figures 3B,C). Finally, 12 lncRNAs were selected for building the optimal prognostic signature of necroptosis-associated lncRNAs, including 8 risk lncRNAs, namely, LINC00460, LINC02773, CHROMR, LINC01094, FLNB-AS1, ITFG1-AS1, LASTR, and LINC01638, and 4 protective lncRNAs, namely, REPIN1-AS1, UBL7-AS1, PINK1-AS, and PVT1. Based on the expression levels of the 12 lncRNAs and the corresponding weighted coefficients, the RS of STAD patients were calculated according to the following formula: RS = (−0.135,699 × REPIN1-AS1) + (−0.143,332 × UBL7-AS1) + (0.065476 × LINC00460) + (0.840948 × LINC02773) + (0.003634 × CHROMR) + (0.150664 × LINC01094) + (0.065846 × FLNB-AS1) + (0.632154 × ITFG1-AS1) + (0.006577 × LASTR) + (-0.177 349× PINK1-AS) + (0.396460 × LINC01638) + (−0.067393 ×PVT1) (Supplementary Table S4).

### Assessment of the 12-NRLs Prognostic Signatures

**FIGURE 4:**
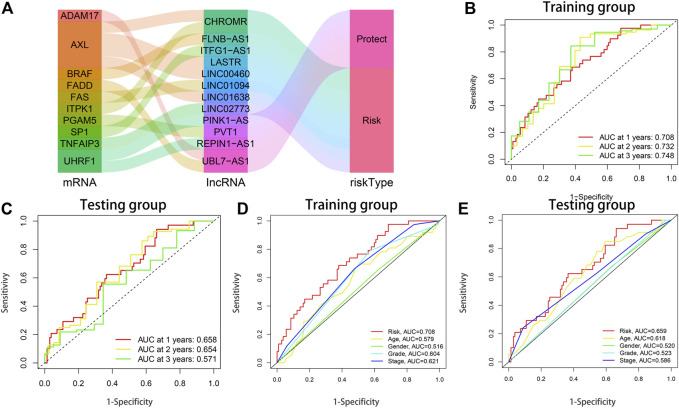
The prognostic performance of the 12-lncRNAs signature in the training and testing sets. **(A)** The Sankey diagram shows the connection degree between the necroptosis-related lncRNAs and necroptosis-associated genes. **(B)** AUC of time-dependent ROC curves at 1-, 2-, and 3-years OS in the training set. **(C)** AUC of time-dependent ROC curves at 1-, 2-, and 3-years OS in the testing set. **(D)** ROC curve analysis of clinicopathological parameters and risk score in the training set. **(E)** ROC curve analysis of clinicopathological parameters and risk score in the testing set.

The 368 STAD samples were randomly allocated into either the training set (*n* = 184) or the testing set (*n* = 184) at a ratio of 1:1. According to the equation above, the clinical RS of each STAD patient was calculated. The median RS of the training set was utilized as the cut-off value to assort both the training and testing sets into the low-risk and high-risk subsets. As exhibited in the scatterplot and risk curve, death cases increase significantly with higher RS (Supplementary Figure S1A–D). Heatmaps were drawn to show the expression outlines of the 12 NRLs in the high-risk and low-risk subsets. Among them, 8 risk lncRNAs were significantly upregulated in the high-risk subset, while 4 protective lncRNAs were significantly downregulated (Supplementary Figure S1E, F). The K-M curves revealed that the OS of patients with a low RS was significantly longer than those with a high RS (training set: *p* < 0.001; testing set: *p* = 0.011) (Supplementary Figure S1G, H). The Sankey diagram not only fully showed the interaction of necroptosis-related lncRNAs and necroptosis-associated genes, but also further illustrated the association between the 12-NRLs risk signature and the OS of STAD patients (Figure 4A). The ROC curve was used to assess the prediction performance of the risk signature for the OS of STAD patients. The AUC values of 1, 2, and 3 years for the training and testing sets reached 0.708, 0.732, 0.748, and 0.658, 0.654, 0.571, respectively. Compared with other clinicopathological features, the AUC of RS was the highest (training set: AUC = 0.708; testing set: AUC = 0.659) (Figures 4B–E). The results indicate that the 12-NRLs signature can prove valuable for patients with STAD.

### Establishment and Assessment of the Prognostic Nomogram Based on the 12-NRLs Signature

**FIGURE 5:**
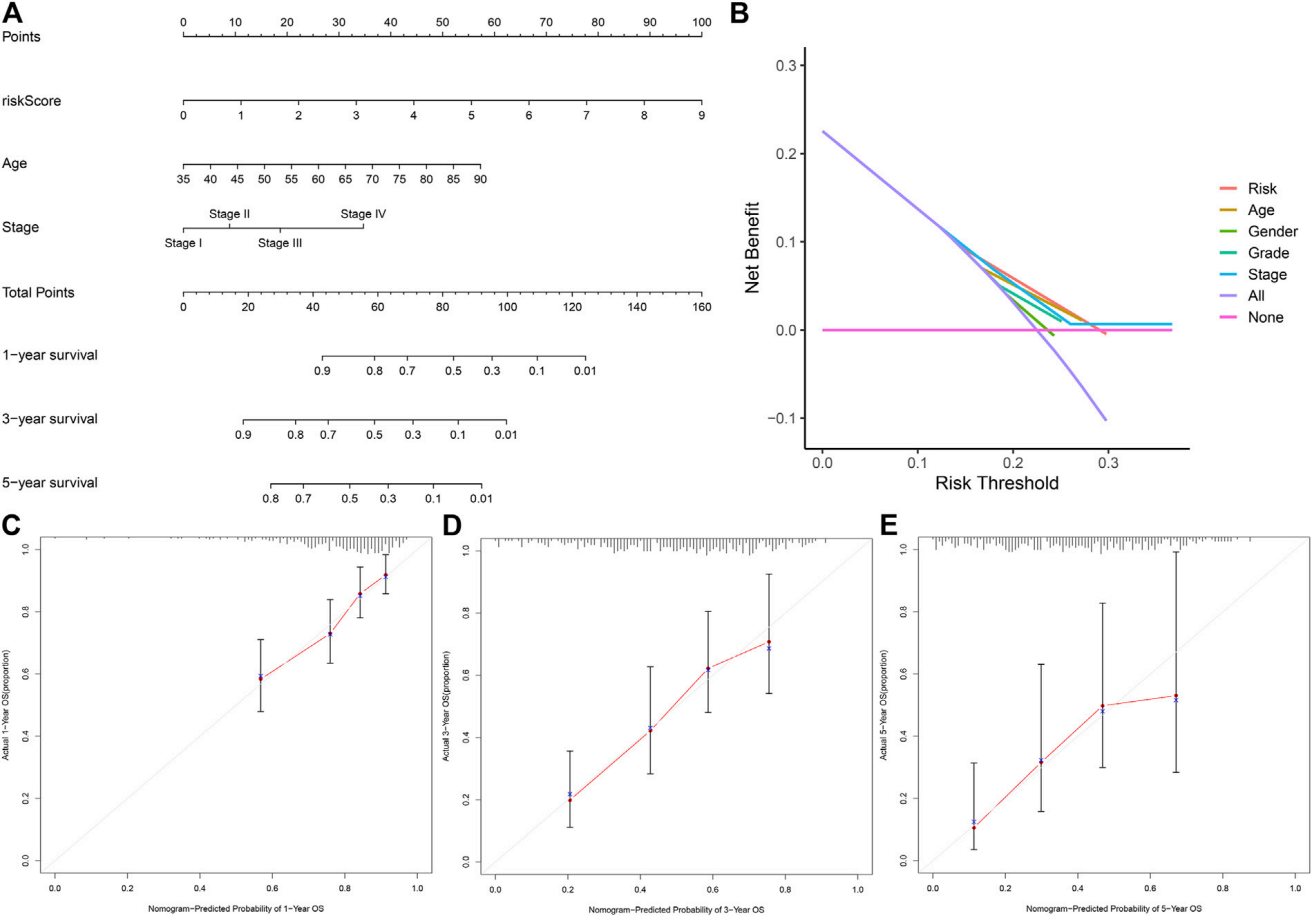
The nomogram for predicting the OS of STAD patients at 1, 3, and 5 years. **(A)** Construction of the nomogram comprising independent prognostic factors (age, stage, and RS). **(B)** DCA curves for assessment of the clinical utility of the nomogram. **(C–E)** The Calibration curves of the nomogram for predicting the probability of OS at 1, 3, and 5 years.

In order to investigate whether the 12-NRLs signature is an independent prognostic predictor for patients with STAD, Cox regression analysis was carried out. According to univariate Cox regression analysis, RS (*p* < 0.001), stage (*p* < 0.001), and age (*p* = 0.002) were significantly correlated with the OS of STAD patients (Supplementary Figures S2A,B). Moreover, multivariate Cox regression further demonstrated that RS (*p* < 0.001; hazard ratio (HR) = 1.665), stage (*p* < 0.001; HR = 1.694), and age (*p* < 0.001; HR = 1.048) were independent risk factors affecting the prognosis of patients with STAD (Supplementary Figures S2C,D). In order to transform the lncRNAs signature into clinical utility, a nomogram involving the independent risk factors (stage, age, RS) was created to estimate and quantify the survival probability of STAD patients after 1, 3, and 5 years (Figure 5A). Additionally, the calibration curves were plotted to verify the predictive capability of the nomogram, the results of which suggested the optimal concordance between the predicted survival probability based on the nomogram and the practical observations (Figures 5C–E). Finally, the DCA curve further confirmed that the nomogram based on the 12-NRLs signature had great clinical applicability in estimating the OS of patients with STAD (Figure 5B).

### Correlation Between the 12-NRLs Prognostic Signature and Clinicopathological Parameters

A Chi-square test was performed to investigate the associations between the 12-NRLs prognostic signature and clinicopathological parameters. The resulting heatmap revealed that the tumor stage (*p* < 0.05), stage M (*p* < 0.05), stage T (*p* < 0.05), and the histological grade (*p* < 0.001) were significantly different in the high-risk and low-risk subsets (Supplementary Figure S3A). Stratified survival analysis was performed to demonstrate the broad applicability of the signature using the following clinicopathological features (age, gender, grade, stage T, stage N, and stage M) (Supplementary Figures S3B–H), which indicated that in the subsets of males and females, >65 years old and ≤65 years old, G1-2 and G3, N 1–3, III-IV, M0, and T 3–4, the OS of patients in the high-risk subset was significantly poorer than those in the low-risk subset. Briefly, all these results illustrate that the signature has good applicability in predicting prognosis.

### Pathway Enrichment Analysis

**FIGURE 6:**
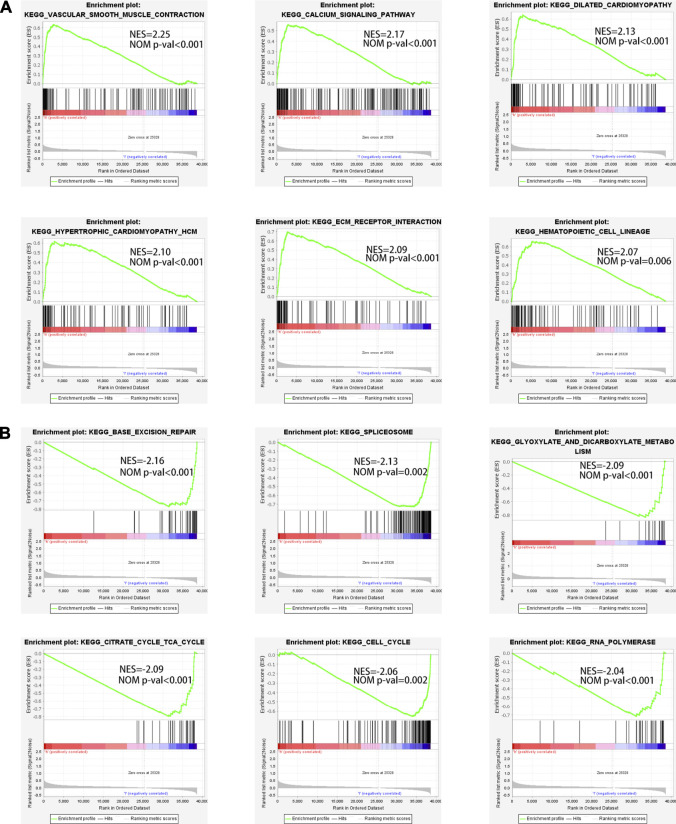
The enriched pathways in high-risk and low-risk subsets obtained using the 12-NRLs signature. **(A)** Top 6 significantly enriched KEGG pathways in the high-risk subset. **(B)** Top 6 significantly enriched KEGG pathways in the low-risk subset.

In order to elucidate the differences of enriched pathways between the low-risk and high-risk subsets, GSEA was adopted for KEGG pathway enrichment analysis. The results revealed that the top 6 pathways substantially enriched in the high-risk subset were vascular smooth muscle contraction, hypertrophic cardiomyopathy HCM, dilated cardiomyopathy, ECM receptor interaction, calcium signaling pathway, and hematopoietic cell lineage (Figure 6A). Base excision repair, glyoxylate and dicarboxylate metabolism, spliceosome, citrate cycle TCA cycle, cell cycle, and RNA polymerase were markedly enriched in the low-risk subset (Figure 6B).

### The Characteristics of TME and Immune Cell Infiltration Between the High-Risk and Low-Risk Subsets

**FIGURE 7:**
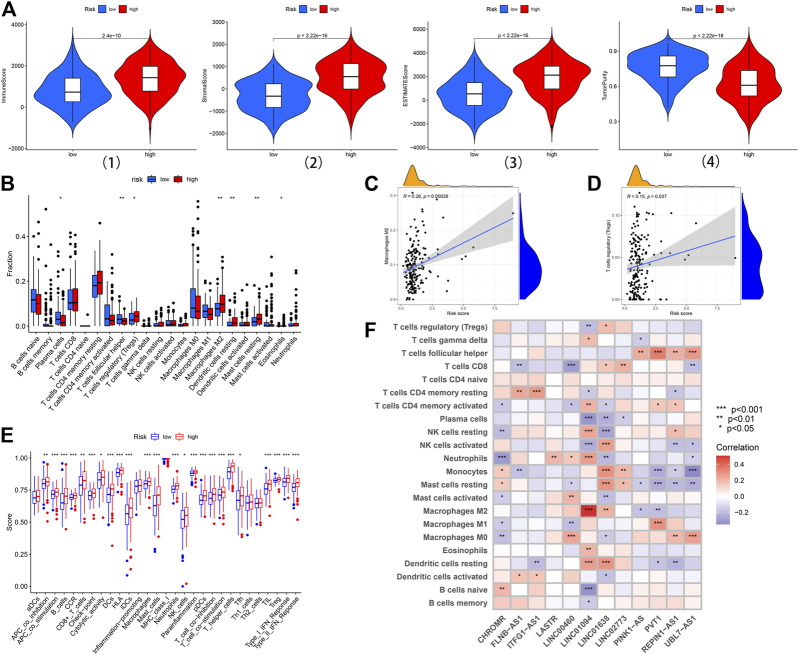
The landscape of immune infiltration in the high-risk and low-risk subsets. **(A)** Comparison of the immune score, stromal score, ESTIMATE score, and tumor purity in the low-risk and high-risk subsets. **(B)** Difference analysis of 22 immune cells infiltration between the high-risk and low-risk subsets. **(C, D)** Correlation analysis between infiltrating level of macrophages M2, and Tregs and risk score. **(E)** Immune functional differences between the low-risk and high-risk subsets based on the ssGSEA scores. **(F)** The correlation analysis between the risk signature and infiltration immune cells. *:*p* < 0.05 **: *p* < 0.01 ***:*p* < 0.001.

In general, TME was composed of the tumor, stromal, and immune cells. TME scores were applied to measure the differences in the extent of infiltrating stromal and immune cells between the low-risk and high-risk subsets via the “ESTIMATE” package. Samples in the high-risk subset had higher immune, stromal, and estimate scores and lower levels of tumor purity (Figure 7A). In order to further understand the significant difference of TME in different risk subsets, the relative expressions of 22 types of common infiltrated immune cells were calculated and compared between the two groups. The results revealed that T cells regulatory (Tregs), macrophages M2, eosinophils, dendritic cells resting, and mast cells resting were significantly upregulated in the high-risk subset; the expression of T cells follicular helper and plasma cells were significantly upregulated in the low-risk subset (Figure 7B). Notably, the infiltrated level of immune‐suppressive macrophages M2 (Cor = 0.26, *p* = 0.00028) and Tregs (Cor = 0.15, *p* = 0.037) was positively correlated with RS, indicating that the poor prognosis of patients in the high-risk subset may be partly due to the immunosuppressive microenvironment (Figures 7C,D). Furthermore, ssGSEA analysis was carried out to assess the enrichment levels of 29 immune-related functions between the two subsets. The results of ssGSEA analysis confirmed conspicuous differences between the two subsets (including APC co-stimulation, B-cells, APC co-inhibition, CCR, CD8^+^ T cells Checkpoint, Cytolytic activity, DCs, Mast cells, Neutrophils, HLA, iDCs, Macrophages, NK cells, T helper cells, pDCs, T cell co−inhibition, T cell co−stimulation, Parainflammation, Tfh, TIL, Type II IFN Response, Type I IFN Response, and Tregs) (Figure 7E). In addition, the relationship between 22 types of common infiltrated immune cells and 12 prognostic lncRNAs was highlighted through a correlation heatmap (Figure 7F). INC01094 displayed a distinct negative correlation with Plasma cells and B cells naive but positively correlated with macrophages M2.

### The Importance of the NRLs-Based Signature in Chemotherapy and Immunotherapy

**FIGURE 8:**
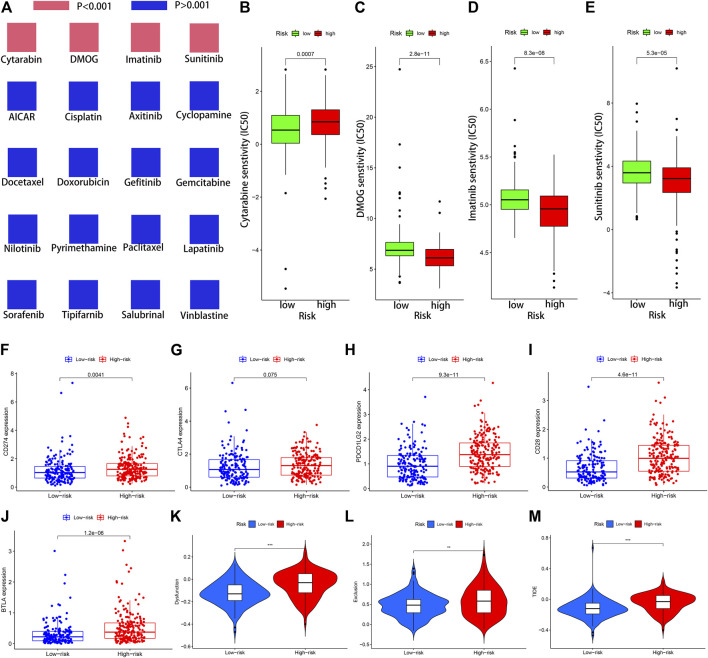
The significance of the NRLs-based signature in chemotherapy and immunotherapy. **(A)** Sensitivity performance of 20 common chemotherapy agents in the high-risk and low-risk subsets. **(B)** Cytarabine, **(C)** DMOG, **(D)** Imatinib, **(E)** Sunitinib. **(F–J)** Scatter plots visualizing markedly differential expression of the immune checkpoint genes CD274, BTLA, CD28, CTLA4, and PDCD1LG2 between the high-risk and low-risk patients. Correlation of risk score and other immune-related prognostic scores. **(K–M)** T cell dysfunction, T cell exclusion, and TIDE score in different risk subsets. *:*p* < 0.05 **:*p* < 0.01 ***:*p* < 0.001.

The IC50 values of 20 conventional chemotherapy agents in low-risk and high-risk subsets were calculated using the “pRRophetic” algorithm to assess the responses of STAD patients to chemotherapy. The two risk subsets had no statistically significant difference in their responses to the 16 chemotherapy agents but significantly different responses to the 4 anticancer drugs (Figure 8A). Moreover, patients in the low-risk subset were particularly sensitive to Cytarabine, which may be applied to SATD patients with a lower RS (Figure 8B). The IC50 values of DMOG, Imatinib, and Sunitinib in the low-risk subset were higher, and these three drugs may be more applicable for patients with a higher RS based on the 12-NRLs signature (Figures 8C–E).

Given the significance of immune checkpoint inhibitor-based immunotherapy, the expression levels of five common immune checkpoint molecules (CD274, BTLA, CD28, CTLA4, and PDCD1LG2) between the low-risk and high-risk subsets were compared to evaluate the responses of STAD patients to immunotherapy. In comparison with STAD patients in the low-risk subset, five common immune checkpoint molecules had a higher expression in the high-risk subset, but the overexpression of CTLA4 was non-significant (*p* = 0.075) (Figure 8F–J). This indicated that STAD patients in the high-risk subset may up-regulate the expression of immune checkpoint genes to mediate immune evasion, which might confer patients’ unfavorable prognosis.

It was reported that TIDE algorithm can be applied to assess patient’ clinical response to ICI therapy. The higher the TIDE score, the greater the likelihood of immune escape, which might mean more limited response to ICI therapy and the shorter survival time. To predict the clinical response to ICI therapy for STAD patients, TIDE analysis was carried out to evaluate the potential efficacy of immunotherapy in different risk subsets. Patients in the high-risk subset had higher TIDE, T cell exclusion, and T cell dysfunction score, indicating that STAD patients in the high-risk subset might less likely to benefit from ICI therapy than in the low-risk subset (Figure 8K–M).

### The Landscape of Somatic Gene Mutations Based on the 12-NRLs Signature

**FIGURE 9:**
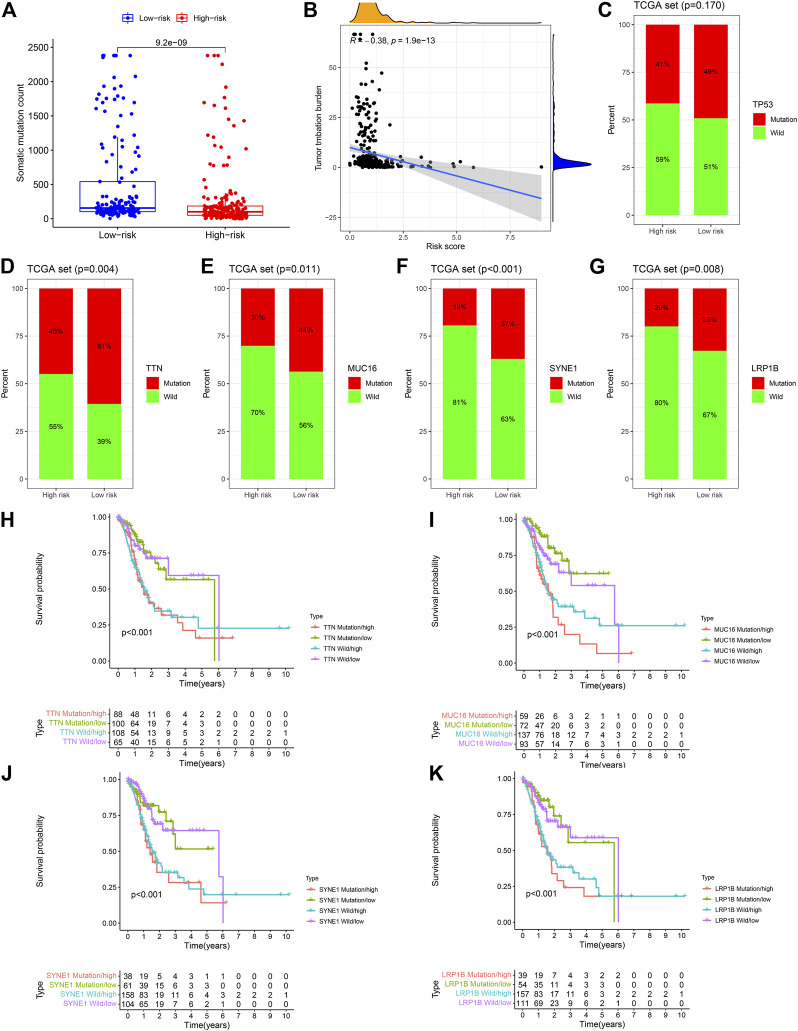
The mutant landscape of the high-risk and low-risk STAD patients. **(A)** Comparative analysis of mutation events between the high-risk and low-risk subsets. **(B)** Mutation events are negatively correlated with RS. **(C–G)** Difference analysis of the top 5 mutant genes between the low-risk and high-risk subsets. **(H–K)** Survival analysis based on the risk classification and mutation status of TTN, MUC16, SYNE1, and LRP1B.

Given the significance of the tumor mutation burden (TMB) affecting the clinical responses of immune checkpoint inhibitors, information on somatic gene mutations of STAD patients was employed to investigate the correlations between TMB and RS. The detailed gene mutation information and regularity of STAD patients are presented in Supplementary Figure S4. Mutational events may be observed in the low-risk subset in comparison to the high-risk subset (*p* < 0.001) (Figure 9A). Spearman’s correlation analysis revealed that RS had a significantly negative correlation to TMB (Spearman’s correlation coefficient = −0.38) (Figure 9B). The mutational frequencies of the top 5 mutant genes between the low- and high-risk subsets were compared through the comparative mutation spectrum analysis (Figures 9C–G). Significant differences between the two risk subsets were observed in TTN, MUC16, SYNE1, and LRP1B. Then, based on the differential gene mutations and risk status, STAD patients were classified into four subsets according to these four genes to perform survival analysis. The subsequent results demonstrated that the OS of the four subsets corresponding to the four genes had significant differences (*p* < 0.001) (Figures 9H–K). The OS of patients in the TTN mutation/low-risk subset was longer than those in the TTN wild/high-risk subset. Similar results were also detected in the MUC16, SYNE1, and LRP1B, indicating that carrying wild-type genes was a hazard factor for the high-risk subset according to the 12-NRLs signature.

## Discussion

As a new type of strictly controlled cell death, necroptosis is mainly regulated by the activation of RIPK1 and RIPK3 and then executed by MLKL (phosphorylation, oligomerization, and membrane translocation), which eventually induces cell death (Cho et al., 2009; He et al., 2009; Zhang et al., 2009; Chen et al., 2014; Wang et al., 2014). Recent research has indicated that the role of necroptosis is paradoxical in anticancer-related biological processes (Zhu et al., 2019). On the one hand, necroptosis can be induced to play a considerable anticancer role when apoptosis is resisted. Smac mimetic can induce necroptosis in caspase-8-deficient colorectal cancer cells, thereby impeding the growth and proliferation of cancer cells in a mouse colorectal cancer model (He et al., 2017). Shikonin suppresses osteosarcoma progression *in vivo* by increasing the activity of necroptosis (Fu et al., 2013). Combining Smac mimetic Birinapant with caspase-8 inhibitor Emricasan can promote necroptosis in myeloid leukemia cells, which may emerge as a promising therapeutic method of AML (Brumatti et al., 2016). However, necroptosis of tumor cells can also facilitate cancer dissemination and metastasis. Necroptosis biomarkers are detected around the necrotic foci of breast cancer tissues in mice and humans, and necroptosis activity enhancement is involved in the aggravation of breast cancer progression and metastasis (Jiao et al., 2018). The invasion and migration capability of head and neck squamous cell carcinoma (HNSCC) cells are enhanced by inducing necroptosis, the level of which is correlated with the survival of HNSCC patients (Li et al., 2020).

Considerable literature has focused on the effect of necroptosis on GC, while studies concerning the effects of NRLs are extremely lacking. Thus, investigating the prognostic value and molecular characteristics of NRLs in STAD is essential for the diagnosis and treatment of GC patients. In this study, NRLs were derived from the TCGA-STAD Transcript data using Pearson correlation analysis. Then, univariate analysis and LASSO regression analysis were employed to determine the most valuable NRLs for STAD prognosis. Eventually, 12 lncRNAs were selected to create the prognostic signature of necroptosis-associated lncRNAs (i.e., REPIN1-AS1, UBL7-AS1, LINC00460, LINC02773, CHROMR, LINC01094, FLNB-AS1, ITFG1-AS1, LASTR, PINK1-AS, LINC01638, and PVT1).

It has been reported that LINC00460 can enhance epithelial-mesenchymal transition (EMT) by promoting peroxidase-1 entrance into the nucleus in HNSCC (Jiang et al., 2019). Moreover, LINC00460 can silence CCNG2 to accelerate GC progression through EZH2/LSD1 epigenetics (Yang et al., 2020). LINC01094 has been demonstrated to enhance the invasion, migration, and EMT capabilities of ovarian cancer cells by adsorbing miR-577 (Xu et al., 2020). Additionally, LINC01094 also promotes the growth and metastasis abilities of glioblastoma cells through sponging miR-126-5p (Li and Yu, 2020). Furthermore, LASTR produced under stress can maintain and accelerate the growth of triple-negative breast cancer (TNBC) cells by regulating the activity of SART3 (De Troyer et al., 2020); PINK1-AS can facilitate Gαi1-driven GC tumorigenesis through sponging microRNA-200a (Lv et al., 2021); LINC01638 is significantly upregulated in both NSCLC tissues and cells and regulated by the transcription factor SP1, which can enhance the proliferation of NSCLC cells and inhibit its apoptosis (Guo et al., 2019).

In order to demonstrate the practical value of the 12-NRLs signature, all STAD samples included were randomly distributed into the training and testing sets at a 1:1 ratio. The AUC values of ROC curves indicated that the signature had an acceptable predictive accuracy in both the training and testing sets (AUC = 1, 2, and 3 years reached 0.708, 0.732, and 0.748 in the training set; AUC = 0.658, 0.654, and 0.571 in the testing set). The signature proved broadly applicable and independent via Cox regression and stratified survival analysis. Calibration and DCA curves were plotted to confirm the predictive capability of the nomogram.

The immune cell infiltration in TME typically evolves along with tumorigenesis and development. In this study, samples in the high-risk subset had higher immune scores and lower levels of tumor purity based on TME scores. Further analysis revealed that the expression of T cells regulatory, macrophages M2, eosinophils, dendritic cells resting, and mast cells resting in the high-risk subset were significantly upregulated, indicating that these abnormal infiltrating immune cells may be correlated with STAD initiation and development. In particular, the infiltrated degree of immune‐suppressive macrophages M2 and Tregs was positively correlated with RS, suggesting that the unfavorable prognosis of patients in the high-risk subset may be partly due to the immunosuppressive microenvironment.

Chemotherapy and immunotherapy accompanied by surgery have become the primary approaches for GC. The sensitivity of 20 common chemotherapy agents in the low-risk and high-risk subsets were compared to guide clinicians in selecting appropriate anticancer drugs for STAD patients. Given the significance of immune checkpoint inhibitor-based immunotherapy, the discrepancies of five immune checkpoint blockade-related molecules between the low-risk and high-risk subsets were examined to assess the sensitivity of STAD patients to immunotherapy. The expressions of five common immune checkpoint molecules were higher in the high-risk subset, revealing that STAD patients in the high-risk subset may up-regulate the expression of immune checkpoint genes to mediate immune evasion, which might confer patients’ unfavorable prognosis. TIDE score had been extensively applied to predict the therapeutic sensitivity with ICI treatment in many solid tumors. TIDE encompassed two underlying mechanisms of tumor immune escape: T cell dysfunction and T cell exclusion (Jiang et al., 2018). Patients in the high-risk subset had distinctly higher TIDE, T cell exclusion, and T cell dysfunction score, indicating that patients in the high-risk subset might possess more limited response to ICI therapy. In addition, TMB has been reported to predict the immunotherapy response of tumor patients (Rizvi et al., 2015; Chalmers et al., 2017). RS was found negatively correlated with TMB in this research, which may be associated with the immunological effects.

In short, the 12-NRLs signature was successfully created to predict the survival of STAD patients in this study. Additionally, investigating the molecular features based on the 12-NRLs risk signature was essential to expand new strategies and ideas for improving the therapy for STAD patients.

## Conclusion

The 12-NRLs risk signature may help assess the prognosis and molecular features of STAD patients and improve the treatment modalities, which can be further applied clinically.

## Data Availability

The datasets presented in this study can be found in online repositories. The names of the repository/repositories and accession number(s) can be found in the article/Supplementary Material.

## Abbreviations

GC: Gastric cancer; LncRNAs: Long non-coding RNAs; NRLs: Necroptosis-related lncRNAs; STAD: Stomach adenocarcinoma; RNA-seq: RNA-sequencing; TCGA: The Cancer Genome Atlas; LASSO: The least absolute shrinkage and selection operator; ROC: Receiver operating characteristic; K-M: Kaplan-Meier; DCA: decision curve analysis; RIPK1/RIPK3:Receptor Interacting Protein Kinase1/3; MLKL: Mixed Lineage Kinase Domain Like Pseudokinase; AML: acute myeloid leukemia; LncRNA: Long non-coding RNA; ncRNAs: non-protein coding RNAs; RS: Risk score; OS: Overall survival; AUC: areas under the curve; TME: Tumor microenvironment; ssGSEA: Single sample Gene Set Enrichment analysis; GSEA: Gene Set Enrichment Analysis; KEGG: Kyoto Encyclopedia of Genes and Genomes; IC50:Half maximal inhibitory concentration; HR: Hazard ratio; Tregs: T cells regulatory; TMB: Tumor mutation burden; HNSCC: Head and neck squamous cell carcinoma; EMT: Epithelial-mesenchymal transition; TNBC: triple-negative breast cancer; TIDE: Tumor Immune Dysfunction and Exclusion; ICI: Immune checkpoint inhibitor; FPKM: Fragments Per Kilobase Million.
