# Supplementary figures and images for: A Necroptosis-Related lncRNA-Based Signature to Predict Prognosis and Probe Molecular Characteristics of Stomach Adenocarcinoma
Source: Front Genet. 2022 Mar 7;13:833928. doi: 10.3389/fgene.2022.833928 (PMC8940523; doi:10.3389/fgene.2022.833928)

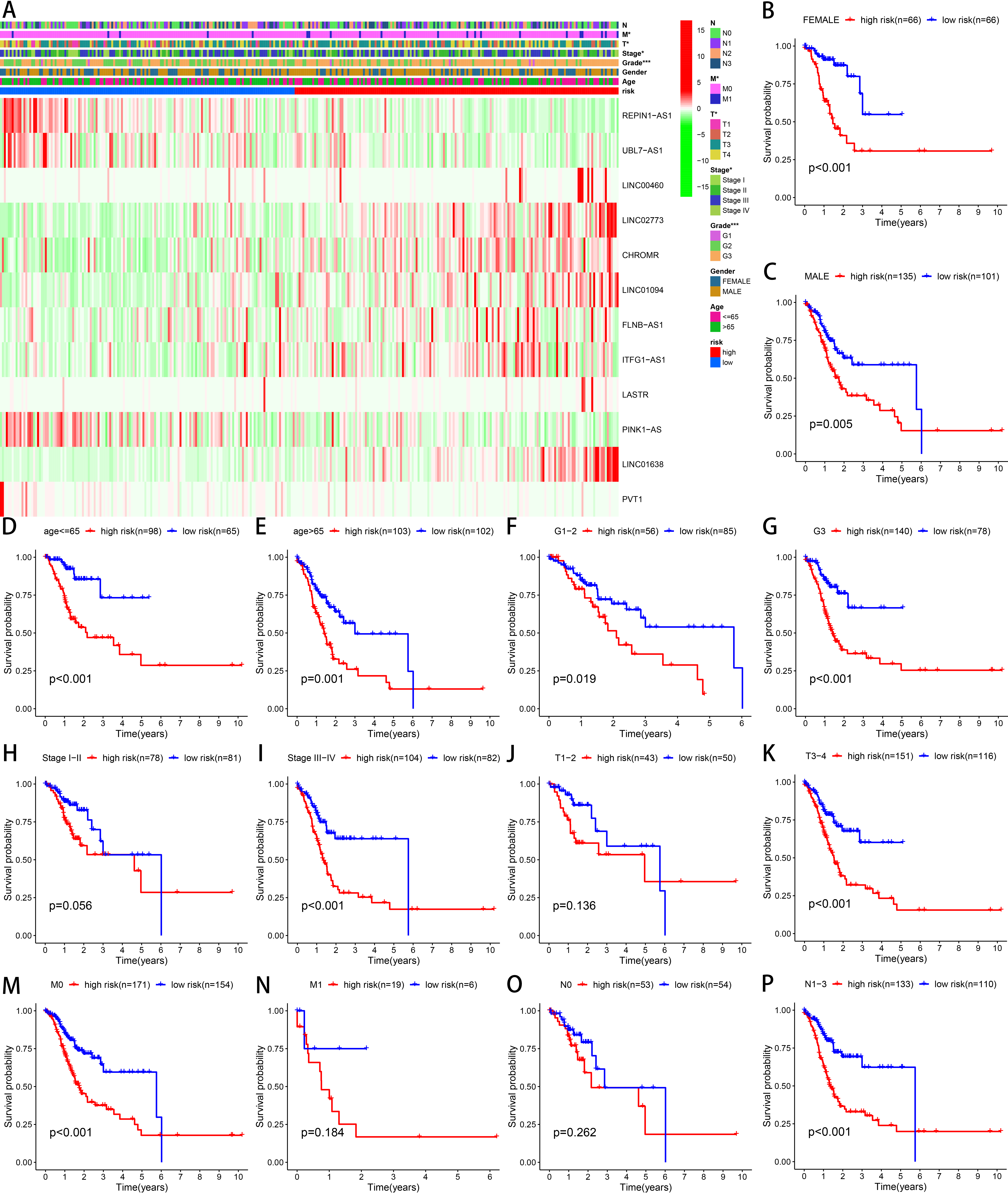

Supplement: Supplementary file 3 [file Image3.TIF]

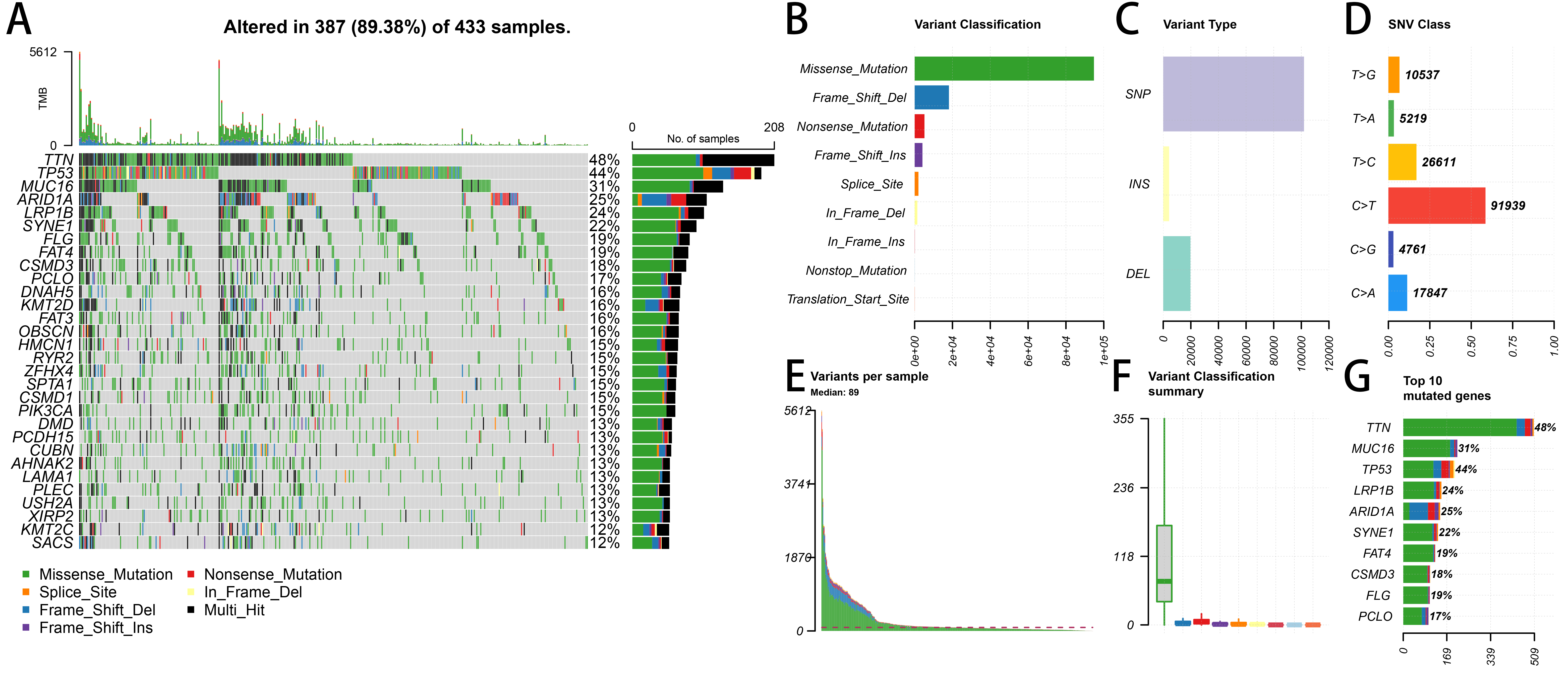

Supplement: Supplementary file 4 [file Image4.TIF]

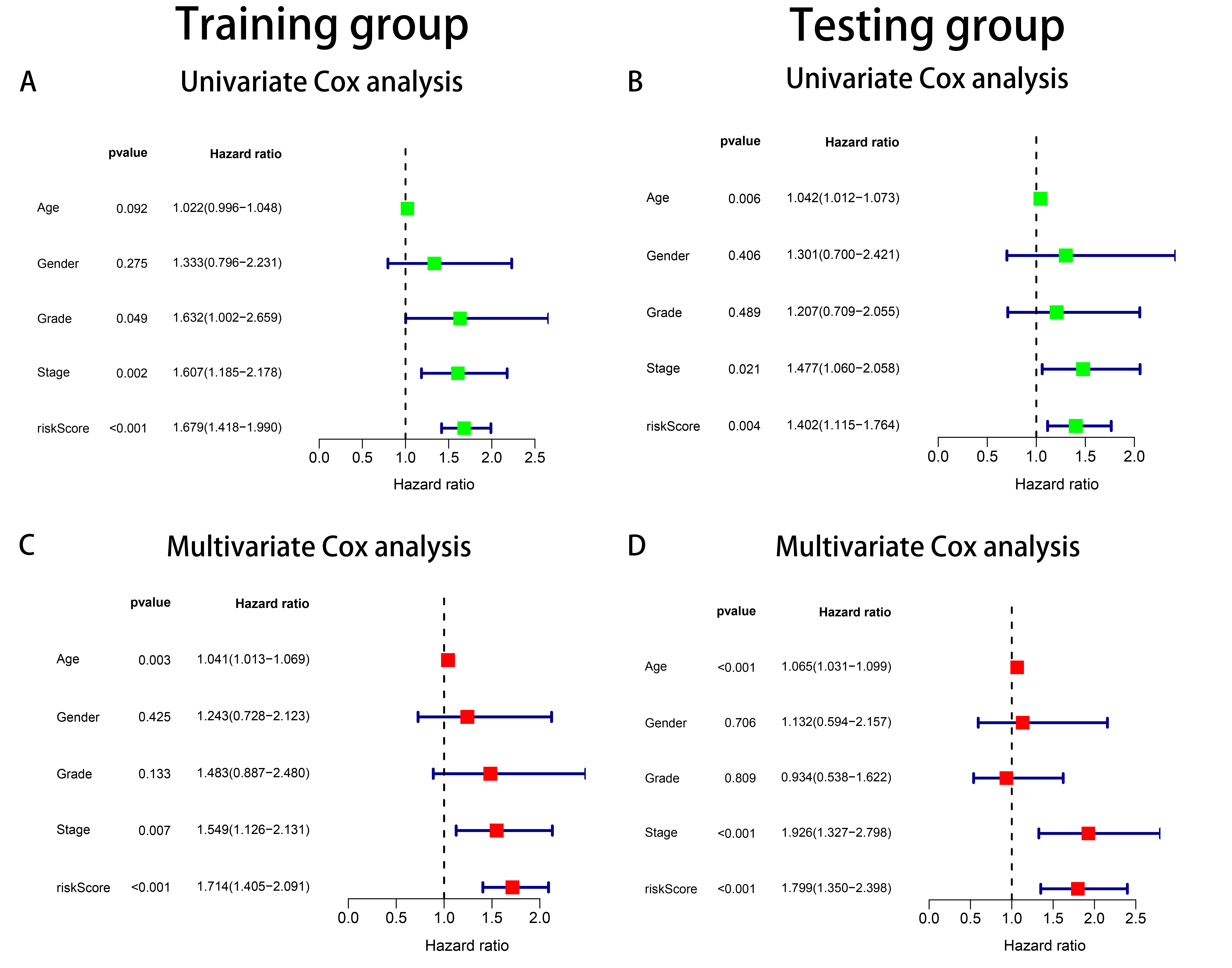

Supplement: Supplementary file 5 [file Image2.TIF]

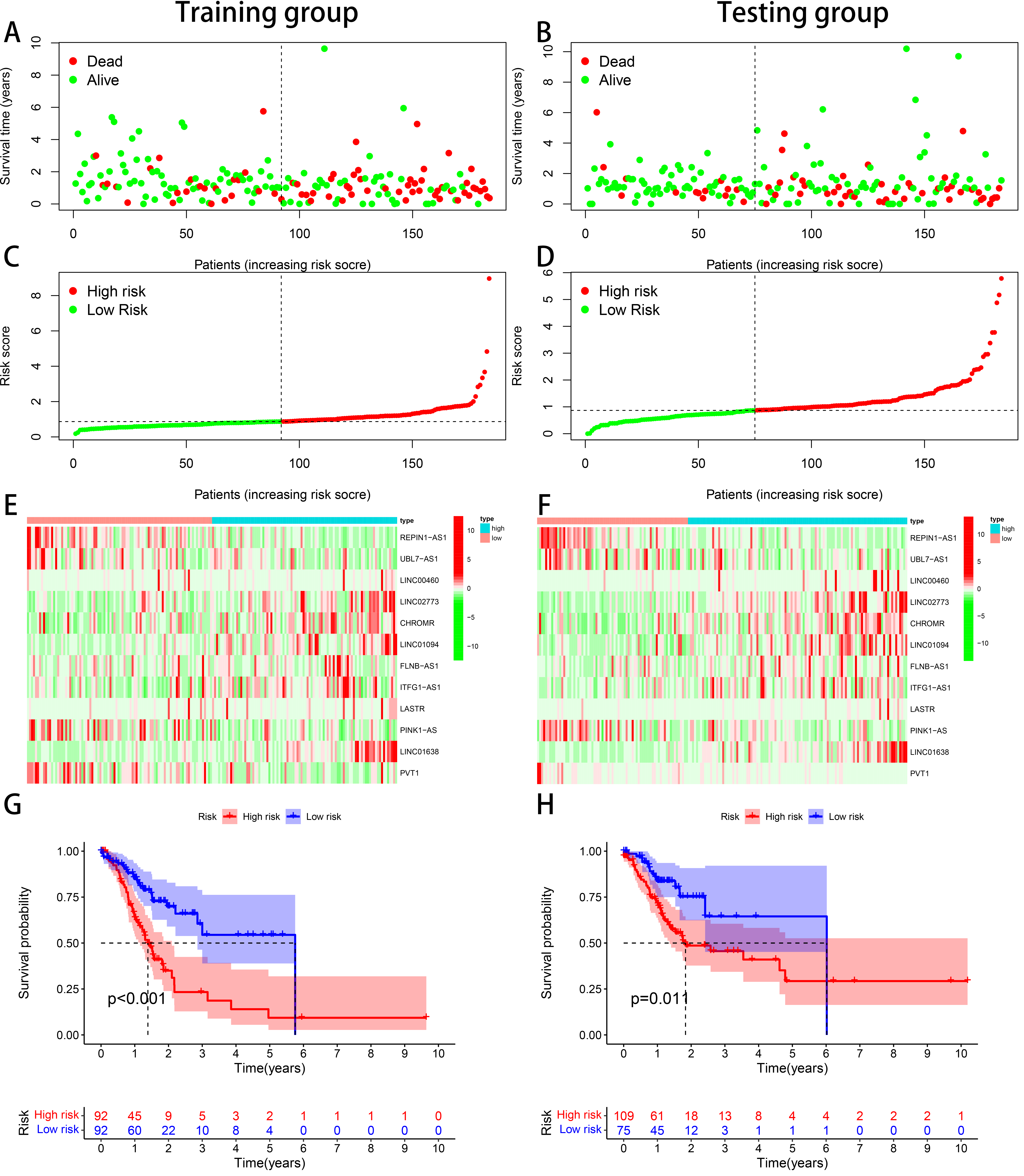

Supplement: Supplementary file 6 [file Image1.TIF]
